# Use of Glucagon-Like Peptide-1 Medications and Modified Medication Use Among Adults — United States, 2025

**DOI:** 10.15585/mmwr.mm7544a1

**Published:** 2026-11-12

**Authors:** Samantha L. Pierce, Lilanthi Balasuriya, Lyudmyla Kompaniyets, Alyson B. Goodman

**Affiliations:** ^1^Division of Nutrition, Physical Activity, and Obesity, National Center for Chronic Disease Prevention and Health Promotion, CDC; ^2^U.S. Public Health Service, Rockville, Maryland.

**Summary**

**What is already known about this topic?**

Glucagon-like peptide-1 (GLP-1) receptor agonists are medications that help regulate appetite, lower blood glucose levels, and promote weight loss. Dispensing of GLP-1s has increased after expanded approvals of these medications for chronic weight management. Compounded GLP-1 medications are not reviewed by the Food and Drug Administration for safety, effectiveness, or quality before they are marketed.

**What is added by this report?**

Based on data from CDC’s Rapid Surveys System collected during July–August 2025, approximately one in 10 adults reported GLP-1 medication use in the past year. Among GLP-1 medication users, approximately one in five reported taking a compounded version. Approximately two in five GLP-1 medication users reported modifying their medication use, often citing cost and availability as barriers.

**What are the implications for public health practice?**

Substantial prevalence of GLP-1 medication use highlights the expanded role of these medications in chronic disease pharmacotherapy. Modifications in medication use reflect, in part, responses to cost- and access-related challenges. Better understanding of real-world medication use, including use of compounded medications and dose and frequency adjustments, is important because it might have implications for safety and effectiveness.

## Abstract

Glucagon-like peptide-1 (GLP-1) receptor agonists are medications that help regulate appetite, lower blood glucose levels, and promote weight loss. GLP-1 medications are approved by the Food and Drug Administration (FDA) for type 2 diabetes treatment, chronic weight management, and treatment of certain other health conditions. Data from CDC’s Rapid Surveys System were used to examine GLP-1 medication use among U.S. adults. During July–August 2025, approximately one in 10 U.S. adults reported taking a GLP-1 medication during the past year. Among GLP-1 users, approximately one in five reported taking a compounded version. Compounded GLP-1 medications are not reviewed by FDA for safety, effectiveness, or quality before they are marketed. Approximately two in five GLP-1 users modified their use of the medication from the way it was prescribed, often citing cost and availability as barriers to prescribed use. Substantial prevalence of GLP-1 medication use highlights the expanded role of these medications in chronic disease pharmacotherapy. Modifications in medication use, particularly among younger adults, reflect, in part, responses to cost- and access-related challenges. Better understanding of real-world medication use, including dose and frequency adjustments, is important because it might have implications for safety and effectiveness.

## Introduction

Approximately 100 million (40%) U.S. adults have obesity, including approximately 22 million (9%) with severe obesity (*1*). Approximately 29 million U.S. adults have diagnosed diabetes. Among these adults, 88% have overweight or obesity, and 67% have high blood pressure or take blood pressure medication, highlighting substantial co-occurrence of chronic diseases among U.S. adults (*2*).

Glucagon-like peptide-1 (GLP-1) receptor agonists are a highly effective class of medications that mimic hormones in the brain, pancreas, and other organs to regulate appetite, lower blood glucose, and promote weight loss. GLP-1 medications are approved by the Food and Drug Administration (FDA) for type 2 diabetes treatment, chronic weight management, and treatment of certain other conditions.*

FDA approvals of GLP-1 medications for chronic weight management, including semaglutide in 2021 and tirzepatide in 2023, resulted in a substantial increase in GLP-1 medication prescriptions (*3*,*4*). However, several challenges have affected accessibility and availability of GLP-1 medications, including high costs, drug shortages, and limited insurance coverage (*5*,*6*). One study during 2022–2024 found that approximately one half of adults with overweight or obesity (but not diabetes) who initiated a GLP-1 medication discontinued use because of cost or insurance-related issues; another 12% were unable to fill prescriptions because of shortages (*6*). During previous drug shortages,^†^ compounded GLP-1 medications became broadly available as alternatives to commercially produced medications. Although compounded versions contain the same active ingredients as FDA-approved GLP-1 medications, they might contain additional, unregulated ingredients because they do not undergo FDA’s review for safety, effectiveness, or quality before they are marketed; they also might be inaccurately marketed to consumers, including misleading advertising and lack of disclosure about possible adverse effects (*7*). Data on use of compounded products and modifications to GLP-1 medication use remain limited. This study used national survey data to characterize GLP-1 medication use among U.S. adults aged ≥18 years.

## Methods

### Data Source

CDC’s Rapid Surveys System (RSS) data are based on two commercially available, online survey panels. (National Opinion Research Center [NORC] at the University of Chicago’s AmeriSpeak Panel and Ipsos’s KnowledgePanel). Panels are based on probability samples of U.S. households and are designed to reflect the civilian, noninstitutionalized U.S. adult population. To account for selection bias and survey nonresponse, sampling weights are created, calibrated to match population totals from the National Health Interview Survey, and applied to produce representative national estimates. The RSS Round 7 survey collected data during July–August 2025. Cumulative response rates of the two panels were 13.3% (AmeriSpeak Panel) and 3.8% (KnowledgePanel); the survey completion rate was 62.6%, totaling 8,118 completed surveys.^§^

### Analyses

GLP-1 medication use was ascertained by responses to the following question, asked of all 8,118 survey respondents: “In the past 12 months, have you taken an oral or injectable medication for diabetes or weight loss, such as Ozempic, Rybelsus, Wegovy, Mounjaro, Zepbound, Saxenda, Victoza, Trulicity, Byetta, or Bydureon BCise? These are also known as GLP-1 medications and might contain semaglutide, tirzepatide, liraglutide, dulaglutide, or exenatide” (Questionnaire, Rapid Surveys System – Round 7). The 976 adults who reported GLP-1 medication use received follow-up questions about use of compounded medications and modifications to GLP-1 medication use (e.g., skipping doses). Weighted prevalence estimates and 95% CIs of GLP-1 medication use were calculated overall and by sex, age group, race and ethnicity, body mass index (BMI) category, insurance type, household income, urbanicity, U.S. Census Bureau region, and presence of selected chronic health conditions. Among GLP-1 medication users, weighted prevalences of compounded GLP-1 medication use, modifications to GLP-1 medication use, and reasons for modifications were examined. Chi-square tests, including pairwise comparisons, were used to examine differences by characteristics; p-values <0.05 were considered statistically significant. Analyses were conducted in SAS-callable SUDAAN (version 11.0.4; RTI International) to account for the complex sampling design. Variances were estimated using Taylor series linearization. CDC National Center for Health Statistics data presentation standards were used to determine reliability of estimates. This activity was reviewed by CDC, deemed not research, and conducted consistent with applicable federal law and CDC policy.^¶^

## Results

### GLP-1 Medication Use by Adult Characteristics

During July–August 2025, approximately one in 10 U.S. adults reported taking a GLP-1 medication during the past year (9.8%) (Table). Prevalence of GLP-1 medication use was higher among females (11.9%) than males (7.6%) and lower among adults aged 18–39 years (4.8%) than among those aged 40–64 years (13.7%) or those aged ≥65 years (11.1%). GLP-1 medication use was higher among persons in a higher BMI category** (e.g., 8.1% of adults with overweight, 15.6% of adults with class 1–2 obesity, and 24.1% of adults with severe [class 3] obesity).

**TABLE:** Number and percentage of adults who reported using a glucagon-like peptide-1 medication in the past year and those who reported using a compounded version, by selected demographic characteristics, obesity category, and selected chronic health conditions — Rapid Surveys System, United States, 2025

Characteristic	Use of any GLP-1 medication in the past year*		Use of compounded GLP-1 medication^†^ among those who used any GLP-1 in the past year	
	Unweighted no.	Weighted %^§^ (95% CI)	Unweighted no.	Weighted %^§^ (95% CI)
**Total**	**976**	**9.8 (9.0–10.7)**	**213**	**22.6 (18.9–26.8)**
**Sex**				
Female	574	11.9 (10.7–13.3)^¶^	144	27.3 (21.9–33.5)^¶^
Male	402	7.6 (6.7–8.7)^¶^	69	14.8 (11.1–19.4)^¶^
**Age group, yrs**				
18–39	198	4.8 (3.8–5.9)^¶^	56	28.0 (19.9–37.8)
40–64	543	13.7 (12.3–15.2)^¶^	125	22.8 (18.5–27.7)
≥65	235	11.1 (9.2–13.3)^¶^	32	18.6 (10.5–30.6)
Race and ethnicity**				
Black or African American	148	11.9 (9.5–14.7)	31	23.1 (14.4–35.0)
White	578	9.6 (8.6–10.7)	132	24.6 (19.4–30.6)
Hispanic or Latino	115	9.8 (7.9–12.1)	23	21.8 (13.3–32.7)
Other and multiple races	124	9.1 (6.8–11.9)	25	13.5 (7.3–22.3)
**BMI category^†^** **^†^**				
Underweight	8	—^§§^	1	—
Healthy weight	115	4.7 (3.6–6.0)^¶^	36	26.9 (18.3–37.6)
Overweight	227	8.1 (6.8–9.5)^¶^	55	28.8 (19.5–40.4)
Obesity (class 1–2)	382	15.6 (13.8–17.6)^¶^	82	20.1 (15.9–25.1)
Severe obesity (class 3)	180	24.1 (20.0–28.6)^¶^	21	13.7 (7.6–23.6)
**Insurance type**				
Private	695	10.6 (9.6–11.6)^¶^	178	25.9 (21.3–31.1)^¶^
Public	245	10.6 (8.7–12.7)^¶^	31	12.5 (7.8–19.6)^¶^
Uninsured	32	2.7 (1.7–4.1)^¶^	4	—
**Household income**				
<100% FPL	142	8.9 (6.8–11.7)^¶^	17	—
100%–<200% FPL	169	9.1 (7.2–11.5)^¶^	33	15.3 (9.0–24.9)^¶^
200%–<400% FPL	248	8.1 (6.9–9.5)^¶^	52	21.2 (15.3–28.6)^¶^
≥400% FPL	417	11.3 (10.1–12.6)^¶^	111	26.9 (21.0–33.8)^¶^
**Urbanicity**				
Metropolitan area	810	9.6 (8.7–10.5)	173	23.6 (19.3–28.4)
Nonmetropolitan area	166	11.6 (9.2–14.5)	40	17.6 (11.6–25.7)
**U.S. Census Bureau region**				
Midwest	230	10.1 (8.7–11.7)	43	23.6 (17.3–31.2)
Northeast	125	8.0 (6.3–10.1)	23	18.0 (10.4–28.0)
South	408	11.4 (10.1–12.9)	93	22.8 (17.9–28.4)
West	189	8.5 (6.7–10.6)	46	24.0 (13.3–39.2)
**Ever had diabetes** ^¶¶^				
Yes	483	47.4 (42.8–52.0)^¶^	59	9.4 (6.6–13.2)^¶^
No	487	5.7 (5.1–6.4)^¶^	153	34.9 (28.7–41.6)^¶^
Ever had sleep apnea***				
Yes	334	23.3 (20.7–26.3)^¶^	63	16.4 (12.2–21.7)^¶^
No	637	8.1 (7.3–9.0)^¶^	148	24.9 (19.9–30.6)^¶^
**Ever had cardiovascular disease** ^†††^				
Yes	173	21.4 (17.9–25.4)^¶^	29	12.0 (7.2–19.5)^¶^
No	800	9.0 (8.2–9.9)^¶^	183	24.3 (20.0–29.1)^¶^

**Abbreviations**: BMI = body mass index; FDA = Food and Drug Administration; FPL = federal poverty level; GLP-1 = glucagon-like peptide-1.

* Data were collected during July–August 2025. Respondents were asked, “In the past 12 months, have you taken an oral or injectable medication for diabetes or weight loss, such as Ozempic, Rybelsus, Wegovy, Mounjaro, Zepbound, Saxenda, Victoza, Trulicity, Byetta, or Bydureon BCise? These are also known as GLP-1 medications and may contain semaglutide, tirzepatide, liraglutide, dulaglutide, or exenatide.”

^†^ Respondents were asked, “Compounded medications include the same active ingredients as popular brand name drugs but are not FDA-approved. They are made by specialized pharmacies and are often used as an alternative when a medication is in short supply. In the past 12 months, have you taken a generic, compounded version of this medication?”

^§^ Weighted to reflect the total population of U.S. adults, based on estimates from quarter 1 of the 2025 National Health Interview Survey Early Release. Row percentages are presented.

^¶^ Statistical significance was based on p-value <0.05 from chi-square test for overall difference.

** Persons of Hispanic or Latino (Hispanic) origin might be of any race but are categorized as Hispanic; all racial groups are non-Hispanic. The other group includes American Indian or Alaska Native, Asian, Middle Eastern or North African, and Native Hawaiian or Pacific Islander.

^††^ BMI was based on self-reported height and weight and categorized as underweight (<18.5 kg/m^2^), healthy weight (≥18.5 kg/m^2^ to <25 kg/m^2^), overweight (≥25 kg/m^2^ to <30 kg/m^2^), obesity (class 1–2: ≥30 kg/m^2^ to <40 kg/m^2^), and severe obesity (class 3: ≥40 kg/m^2^).

^§§^ Dashes indicated that estimate is not shown because it does not meet National Center for Health Statistics standards of reliability.

^¶¶^ Respondents were asked, “Not including prediabetes or gestational diabetes, has a doctor or other health professional ever told you that you had diabetes?”

*** Respondents were asked, “Have you ever been told by a doctor or other health professional that you had sleep apnea?”

^†††^ Respondents were asked, “Have you ever been told by a doctor or other health professional that you had cardiovascular disease, including coronary artery disease, angina, a stroke, or a heart attack?”

### GLP-1 Medication Use by Chronic Health Conditions 

GLP-1 medication use prevalence also differed by reported presence of chronic health conditions. For example, compared with persons without each condition, GLP-1 medication use was more common among those reporting ever having diabetes (47.4% versus 5.7%), sleep apnea (23.3% versus 8.1%), or cardiovascular disease (21.4% versus 9.0%) (Table). Most GLP-1 users had overweight (25.2%) or obesity (53.8%) (Supplementary Table). Approximately one half of GLP-1 medication users reported ever having had each of the following conditions: prediabetes (59.0%), hypertension (56.2%), and high cholesterol (49.1%).

### Use of Compounded GLP-1 Medications

Approximately one in five GLP-1 medication users (22.6%) reported taking a compounded version of the medication; an additional 8.8% did not know whether their GLP-1 medication was compounded (Supplementary Table). Prevalence of compounded GLP-1 medication use was significantly higher among females (27.3%) than among males (14.8%) and among those with private health insurance (25.9%) than among those with public health insurance (12.5%) (Table).

### Modification of GLP-1 Medication Use

Overall, approximately two in five GLP-1 medication users (44%) reported modifying their GLP-1 medication use from the way it was prescribed; skipping doses (28%) was the most common modification (Figure 1). Compared with adults aged ≥65 years, modifying GLP-1 medication use was significantly more prevalent among those aged 18–39 years (54% versus 30%); for example, compared with older adults, those who were younger more often delayed prescription fills (37% versus 6%). Modification of GLP-1 medication use was also more prevalent among adults taking compounded GLP-1 medications (62%) than among those not taking compounded GLP-1 medications (36%). Among GLP-1 medication users who modified their medication use, 38% cited cost and 36% availability as reasons; 58% of adults aged 18–39 years cited cost as a barrier, compared with 20% of adults ≥65 years (Figure 2). Approximately 12% of GLP-1 medication users reported reducing or stopping taking their medication due to side effects or symptoms.

**FIGURE 1:**
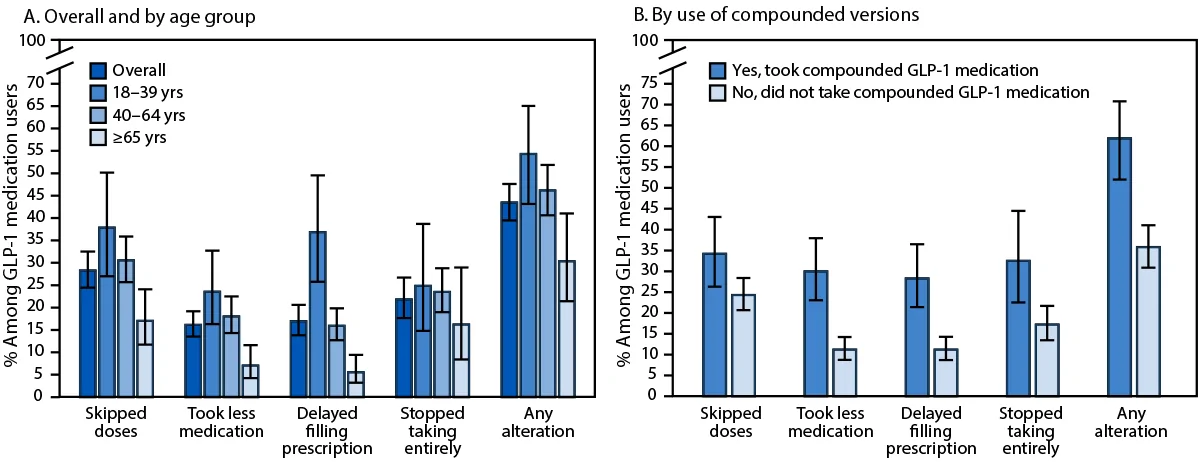
Prevalence*^,^^†^ of respondents who reported modifying use of glucagon-like peptide-1 medications among medication users, by (A) age group and (B) use of compounded versions — Rapid Surveys System, United States, 2025^§^ **Abbreviation**: GLP-1 = glucagon-like peptide-1. * With 95% CIs indicated by error bars. ^†^ p-values <0.05 were considered statistically significant. Overall differences based on chi-square tests were observed for skipped doses, took less medication, delayed filling prescription, and any modification. ^§^ Data were collected during July–August 2025.

**FIGURE 2:**
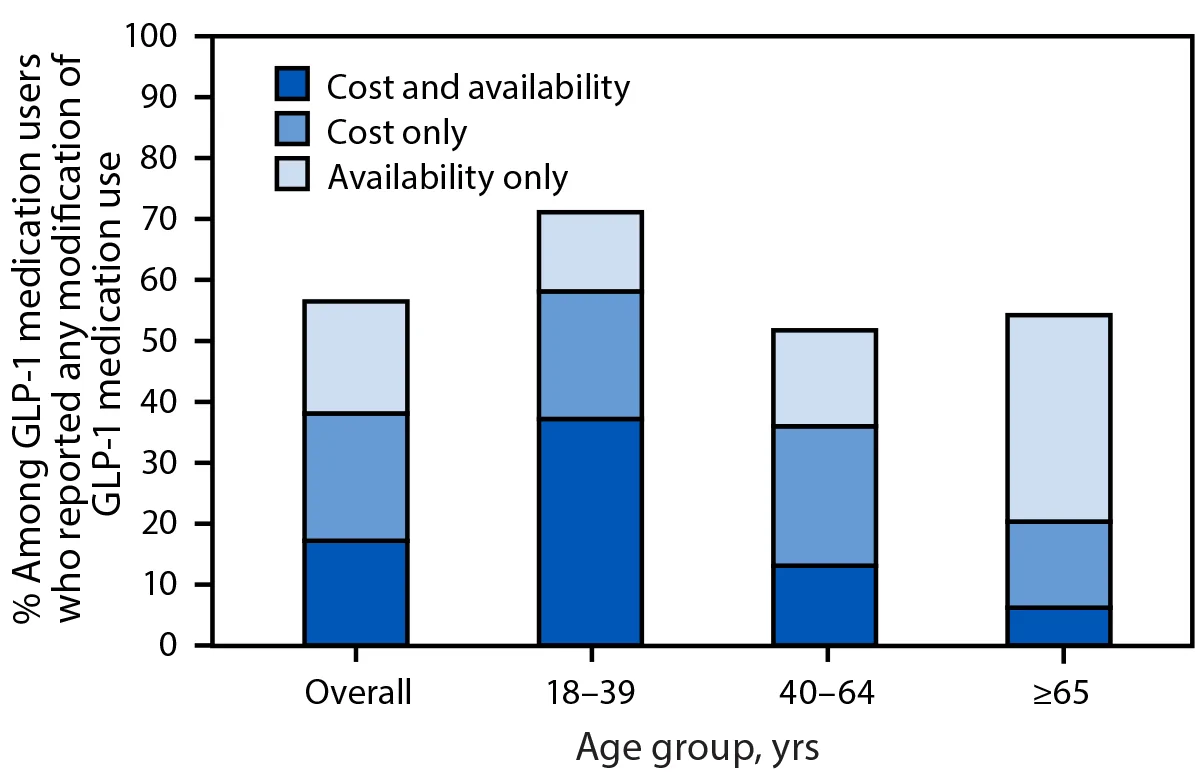
Reported reasons for modifying use of glucagon-like peptide-1 medications, by age group — Rapid Survey System, United States, 2025* **Abbreviation**: GLP-1 = glucagon-like peptide-1. * Data were collected during July–August 2025.

## Discussion

Among a nationally representative sample of U.S. adults in 2025, approximately one in 10 reported taking a GLP-1 medication during the past year, including approximately one half of adults with diabetes and approximately one in five of those with obesity (any class). This suggests substantial population-level use of these medications that help manage excess weight, diabetes, and related conditions.

Most GLP-1 medication users in this study (80%) had overweight or obesity; the remaining 20% could include persons with diabetes but not excess weight, GLP-1 medication users whose BMI had decreased to the healthy weight category, or those taking GLP-1 medications for off-label use. GLP-1 medication use was common among adults with diabetes, sleep apnea, or cardiovascular disease, consistent with findings from a prior online survey and current FDA-approved indications for GLP-1 medications. Further, a large proportion of GLP-1 medication users had prediabetes, hypertension, and high cholesterol. GLP-1 medications might improve multiple components of metabolic health through blood glucose control, reduction of weight or inflammation, or other mechanisms (*8*).

Approximately one in five GLP-1 medication users reported taking compounded formulations. Compounded GLP-1 medications are often more accessible and affordable than are commercially produced formulations, possibly because of insurance coverage restrictions or other reasons (*5*). However, concerns regarding the safety, quality, and effectiveness of compounded GLP-1 medications have been expressed by FDA and the American Diabetes Association. Analyses of FDA adverse event reports have identified disproportionately higher reporting of preparation errors, contamination, and manufacturing issues for compounded products compared with noncompounded products (*9*). Further, approximately 9% of GLP-1 medication users in this study were unsure whether their GLP-1 medication was compounded, potentially reflecting challenges in distinguishing compounded from FDA-approved products. In a recent assessment of websites selling compounded GLP-1 medications, approximately 40% stated or implied the drugs were FDA approved and approximately 80% used brand names in their advertising, which is a violation of FDA regulatory standards (*7*). Given the substantial use of compounded GLP-1 medications, understanding the safety and quality of compounded GLP-1 medications is an important public health consideration.

Modifications in medication use were common among GLP-1 medication users (reported by approximately 40%). Cost and lack of availability were common reasons for modifications; there might be others that were not captured. Adults aged <40 years were more likely than were those aged ≥65 years to report modifications to GLP-1 medication use, including delaying prescription fills, and to cite cost as a barrier. Several factors might contribute to age-related differences, including variations in insurance coverage for GLP-1 medications and reasons for GLP-1 medication use. Nonetheless, across all age groups, ≥50% of GLP-1 medication users who reported modifications cited cost or availability as reasons. This might signal an opportunity for clinicians and health care payers to better understand patient experiences with GLP-1 medications, including barriers to access and other potential reasons for modifying medication use, such as side effects. Given the frequency of reported modifications, additional evidence of the safety and effectiveness of these real-world practices could help guide the development and implementation of clinical guidelines and support appropriate medication use.

### Limitations

The findings in this report are subject to at least three limitations. First, online panel surveys have low cumulative response rates, which could affect generalizability; CDC adjusts for nonresponse through calibration and weighting of RSS data to validated national surveys, such as the National Health Interview Survey. Second, online panel surveys might be less accessible to persons without reliable internet or computer access; however, one of the panel providers offered telephone interviews for respondents who preferred that option. Finally, measurement error from response or recall biases was possible. Cognitive interviewing among a separate sample of adults indicated that respondents generally understood and were able to report on overall and compounded GLP-1 medication use.

### Implications for Public Health Practice

Substantial prevalence of GLP-1 medication use highlights the expanded role of these medications in chronic disease pharmacotherapy (*10*). Modifications in GLP-1 medication use reflect, in part, responses to cost- and access-related challenges. Future research could evaluate the implications of real-world medication use, including dose and frequency adjustments, on safety and effectiveness.

## Footnotes

* GLP-1 medications are approved by FDA to improve glycemic control in adults with type 2 diabetes; for chronic weight management in adults with body mass index (BMI) ≥30 kg/m^2^ (obesity) or ≥27 kg/m^2^ and a weight-related health condition; for moderate to severe obstructive sleep apnea in adults with obesity; to reduce risk for major adverse cardiovascular events in adults with cardiovascular disease and overweight, obesity, or diabetes; and to treat serious liver disease in adults with moderate to advanced fibrosis.

† The tirzepatide shortage was declared resolved on December 19, 2024. The semaglutide shortage was declared resolved on February 21, 2025. After medication shortages are resolved, bulk compounding of these medications is no longer permitted by FDA. FDA clarifies policies for compounders as national GLP-1 supply begins to stabilize | FDA

§ The cumulative response rate was the product of the panel recruitment rate, the panel retention rate, and the study-specific survey completion rate for panel 1, and the product of the panel recruitment rate, panel profile rate, and the study specific survey completion rate for panel 2. NCHS Surveys System Quality Profile, Round 7: Data collected July–August 2025

¶ 45 C.F.R. part 46.102(l)(2), 21 C.F.R. part 56; 42 U.S.C. Sect. 241(d); 5 U.S.C. Sect. 552a; 44 U.S.C. Sect. 3501 et seq.

** BMI was based on self-reported height and weight and categorized as follows: underweight (BMI <18.5 kg/m^2^), healthy weight (BMI ≥18.5 kg/m^2 ^to <25 kg/m^2^), overweight (BMI ≥25 kg/m^2^ to <30 kg/m^2^), obesity (class 1–2: BMI ≥30 kg/m^2^ to <40 kg/m^2^), and severe obesity (class 3: BMI ≥40 kg/m^2^).
